# Nuclear expression of VDR and AHR is mutually exclusive in glandular cells in endometriosis

**DOI:** 10.1007/s00418-021-02005-9

**Published:** 2021-06-21

**Authors:** Francesco De Pascali, Livio Casarini, Christina Kuhn, Manuela Simoni, Sven Mahner, Udo Jeschke, Viktoria von Schönfeldt

**Affiliations:** 1 Department of Obstetrics and Gynaecology, Innenstadt, Ludwig-Maximilian University of Munich, Maistrasse 11, 80337 Munich, Germany; 2 Department of Biomedical, Metabolic and Neural Sciences, Unit of Endocrinology, University of Modena and Reggio Emilia, Modena, Italy; 3 Center for Genomic Research, University of Modena and Reggio Emilia, Modena, Italy; 4 Azienda USL Di Modena, Modena, Italy; 5 Division of Gynecological Endocrinology and Reproductive Medicine, Department of Gynecology and Obstetrics, Ludwig-Maximilians-University, Marchioninistrasse 15, 81377 Munich, Germany

**Keywords:** Immunohistochemistry, Endometriosis, VDR, AHR, Nuclear receptors

## Abstract

The vitamin D receptor (VDR) and aryl hydrocarbon receptor (AHR) are two nuclear receptors that exert their effects by binding with ligands and forming a molecular complex. These complexes translocate to the nucleus and activate the expression of a series of genes which have a response element to VDR or AHR. Both receptors have been identified in the pathogenesis of endometriosis, a common disease characterized by the formation of endometrium-like tissue in ectopic zones. Despite numerous therapies, there is no definitive cure for endometriosis at the pharmacological level. Our study aims to describe the location and the expression of VDR and AHR at the protein level. For this purpose, an evaluation was performed using tissue from the three normal phases of the endometrium (proliferative, early, and late secretory) and in endometriosis by immunohistochemistry, using anti-VDR and anti-AHR antibodies. We demonstrate that in the nuclei of glandular cells in endometriosis, the expression of VDR and AHR is mutually exclusive—when the expression of one receptor is high, the other one is low—suggesting a possible target in the treatment of endometriosis. We also identify a significant change in the expression of glandular cytoplasmic AHR between the proliferative and late secretory endometrium.

**Supplementary Information:**

The online version contains supplementary material available at 10.1007/s00418-021-02005-9.

## Introduction

The vitamin D receptor (VDR) and aryl hydrocarbon receptor (AHR) are two nuclear receptors which have been associated with endometriosis (Sayegh et al. 2014; Rier et al. 1993). Endometriosis is a common gynecologic disease that affects about 10% of all women in the reproductive phase of their lives (Missmer and Cramer 2003). The disorder is defined as the growth of endometrial tissue outside the cavum uteri, and it is postulated to invoke a chronic inflammatory reaction (Agic et al. 2007). Endometriosis shares several similarities with malignant diseases, such as reduced apoptosis, invasion of endometrial cells into adjacent organs (bowel, bladder), increased angiogenesis (Varma et al. 2004), and recurrence (Donnez et al. 2002).

Vitamin D and its receptor (VDR) regulate numerous physiological and pharmaceutical processes, including bone and calcium metabolism, cellular growth and differentiation, immunity, and cardiovascular functions (Nagpal et al. 2005; Choi and Makishima 2009). Numerous in vitro and in vivo studies have shown that vitamin D is a potent inhibitor of cellular proliferation in a wide range of cell types, including carcinomas of the breast, prostate, colon, skin, and brain, myeloid leukemia cells, and others (Guyton et al. 2003; Ravid and Koren 2003; Koeffler et al. 2005). Furthermore, VDRs and enzymes involved in the synthesis and degradation of vitamin D have been identified in many tissues, suggesting a role for vitamin D in the regulation of normal cellular growth at a local level (Holick 2003; Berger et al. 1988). The human endometrium is a steroid hormone-dependent tissue displaying complex cellular regulation mediated by nuclear receptors. Stromal endometrial cells were shown to upregulate VDR and the active form of 1α-hydroxylase in early pregnancy versus cycling endometrium (Vigano, 2006) independently of the menstrual cycle phase. The endometrium is also a site of 1,25(OH)2D extrarenal synthesis and a target of 1,25(OH)2D activity through gene regulation and immunomodulation (Bagot et al. 2000; Lemire et al. 1995). Dysregulation of VDR expression and activity in the endometrium consequently leads to a pathological state. The dysregulation of the VDR pathway in the eutopic endometrium of women affected by endometriosis was studied by Agic et al. (2007). The authors documented an increase in 1α-hydroxylase mRNA expression and a tendency for elevated 24-hydroxylase expression in the endometrium of women with endometriosis compared with controls. This elevation in VDR, 1α-hydroxylase, and 24-hydroxylase mRNA expression in the endometrium of women with endometriosis suggests an active role of the VDR pathway in the pathogenesis of endometriosis.

The aryl hydrocarbon receptor (AHR) is a cytosolic ligand-activated transcription factor that is involved in drug and xenobiotic metabolism (de Tomaso Portaz et al. 2015). The AHR is highly expressed in multiple organs and tissues and plays an important role in cellular homeostasis (Safe et al. 2013). The activation of AHR leads to the formation of an active transcription factor heterodimer with the AHR nuclear translocator (ARNT), and induces expression of a group of genes called the [*Ah*] gene battery (Nebert et al. 2000). Although the precise mechanism of AHR action is still unknown, it has been shown in the endometrium that the agonist-activated AHR/ARNT heterodimer is directly associated with estrogen receptor-α (ER-α) and ER-β, resulting in the recruitment of unbound ER and the coactivator p300 to estrogen-response gene promoters, which leads to activation of transcription and estrogenic effects. In estrogen target tissues such as the endometrium, this mechanism leads to the promotion of cellular proliferation (Ohtake et al. 2003), suggesting a link between AHR activity and endometriosis pathophysiology.

Based on the aforementioned data from the literature, many studies have suggested the direct involvement of VDR or AHR in the pathogenesis and/or modulation of endometrial lesions. Accordingly, we may speculate that both receptors might be upregulated, exerting a concerted action in modulating proliferative signals, in these pathological tissues. Therefore, the aim of this study was to describe the relative expression of these two nuclear receptors in normal endometrial tissues versus ovaries of women diagnosed with ovarian endometriosis (thus presenting infiltration of the endometrium in the ovary). The comparison of VDR or AHR expression in such tissues is based on the nature of endometriosis, which is defined as “the presence of endometrium-like tissue outside the uterus” (Zondervan et al. 2020). A combined analysis of the possible changes in the relative expression of VDR and AHR is currently missing and could provide more insight into the pathophysiological description of endometriosis.

## Materials and methods

### Tissue samples

This study was approved by the Medical School Ethical Committee, Ludwig Maximilian University (LMU), Munich, Germany. Informed consent was obtained from the patients. All data have been anonymized. Samples of human endometrial tissue were obtained from 45 premenopausal, non-pregnant patients undergoing gynecological surgery for benign diseases either by dilatation and curettage or hysterectomy. All women had a normal and regular menstrual cycle with no hormone treatment for 3 months prior to surgery. All pathological and hyperplastic endometrial samples were excluded from this study. Endometrial samples were collected and the order of access to the clinics was registered. After preparation of the slides, samples were classified according to clinical history and histological dating into proliferative (days 1–14), early secretory (days 15–22), and late secretory phase (days 23–28). The first 15 samples per group were chosen at random, considering the patients’ chronological order of accession to the clinics as a unique selection criterion. The ovaries of eight patients diagnosed with ovarian endometriosis were randomly selected from the archives of the Department of Obstetrics and Gynecology of LMU.

### Immunohistochemistry

Formalin-fixed paraffin-embedded sections (3 µm) were dewaxed in xylol, rehydrated in a descending ethanol gradient, and subjected to epitope retrieval in a pressure cooker using sodium citrate buffer (pH 6.0). After returning to room temperature, tissue was blocked with 3% ${\mathrm{H}}_{2}{\mathrm{O}}_{2}$ in methanol (20 min) for endogenous peroxidase activity. Nonspecific binding of the primary antibodies was blocked using the appropriate blocking solution. Sections were then incubated with primary antibodies. The salient features of the antibodies used are presented in Table 1.

**Table 1:** Antibodies used for the study

Antibody (AB)	AB Incubation conditions	Blocking solution	Blocking conditions
VDR monoclonal (mouse IgG2a)	1:100 in PBS; 1 h at room temperature	Power Block (BioGenex, Fremont, CA, USA)	3 min
AHR polyclonal (rabbit IgG)	1:200 in PBS; 16 h at 4 °C	Reagent 1 (Polymer kit, Zytomed System, Berlin, Germany)	5 min

**Fig. 1:**
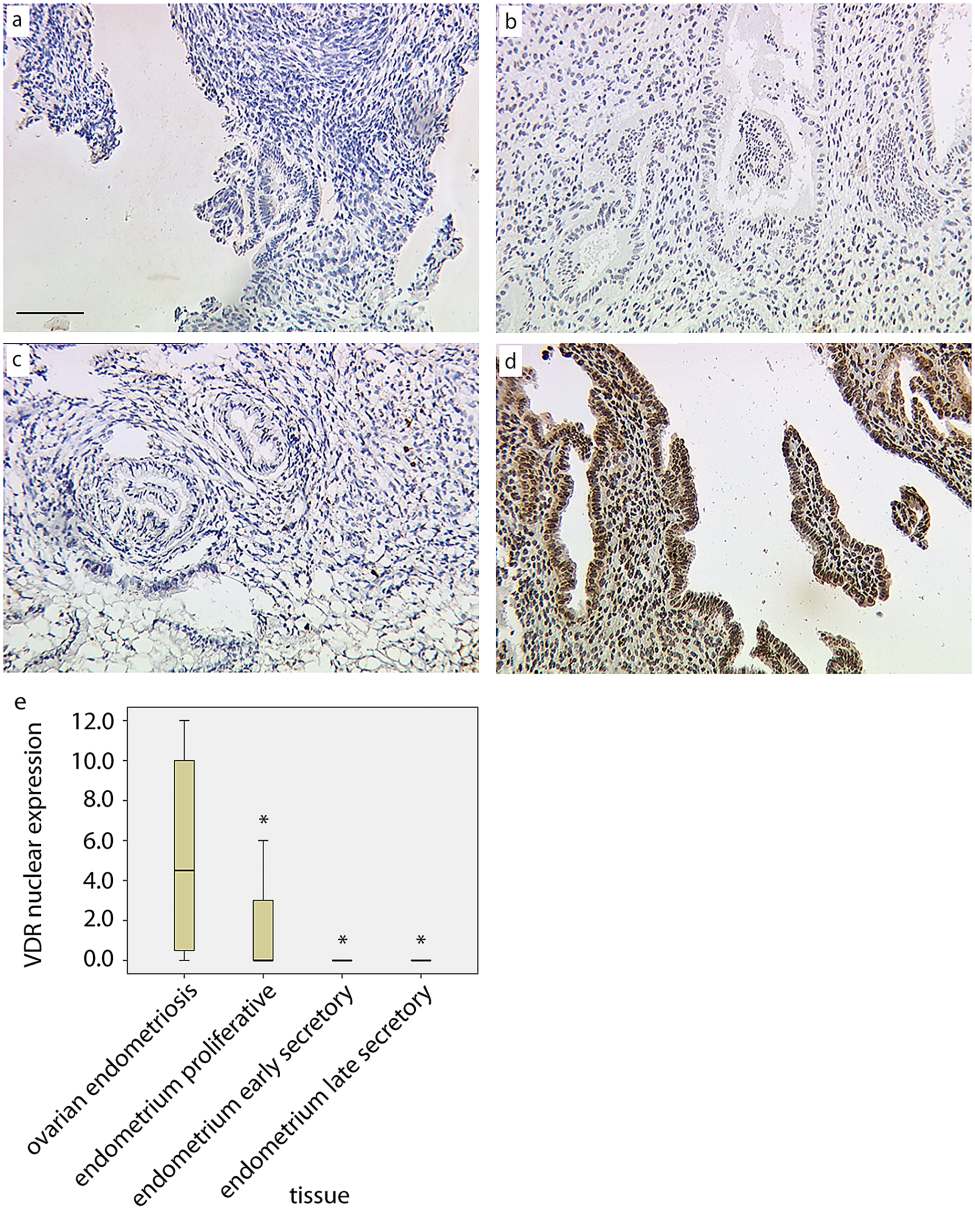
Expression of VDR in the nucleus of endometrial glandular cells from proliferative (**a**), early secretory (**b**), and late secretory (**c**) endometrium as well as from ovarian endometrial tissue (**d**). Box and whiskers plot (**e**) representing the distribution of the IRS scoring for the different tissues analyzed (*significantly different versus “ovarian endometriosis”, Kruskal–Wallis test, *p* < 0.05). Bar = 100 µm

Staining was performed using the Vectastain Elite ABC kit (Vector Laboratories, Burlingame, CA, USA) for anti-VDR antibody, and ZytoChem Plus HRP Polymer Kit (Zytomed Systems, Berlin, Germany) for anti-AHR antibody, according to the manufacturers’ protocol. Substrate and chromogen (3,3′-diaminobenzidine [DAB], Dako, Glostrup, Denmark) were finally added to the slides, which were then counterstained with Mayer’s acidic hematoxylin and covered. Placental tissue (both VDR and AHR) was used for positive control staining (Fig. 1). The sections were examined by two independent observers using a Leitz Diaplan microscope (Leitz, Wetzlar, Germany).

### Statistical evaluation

Ten fields per slide were examined using a semi-quantitative immunoreactivity score (IRS) (Remmele and Stegner 1987). IRS measures the intensity and distribution of antigen expression and is calculated by multiplying the percentage of positively stained cells (0: no staining; 1: < 10% of the cells; 2: 11–50%; 3: 51–80%; 4: > 80%) by the intensity of staining (0: none; 1: weak; 2: moderate; 3: strong). Each field in a given sample was considered as a single observation for each observer (thus two values were produced by the two observers for each of the ten fields). Twenty values per sample slide were reported as replicates for the statistical evaluation. IBM SPSS version 20 software for Windows (IBM Corp., Armonk NY, USA) was used for data collection and processing and for analysis of statistical data. Values with *p* < 0.05 were considered statistically significant. The Mann–Whitney *U* test was used for the evaluation of two independent groups. Spearman’s correlation was used to evaluate correlations of two independent groups.

## Results

### VDR expression in glandular epithelial tissue of normal endometrium and in endometriosis

In the three phases of the endometrium (proliferative, early, and late secretory) we were unable to observe expression of VDR in the nuclei of the glandular cells of the proliferative endometrium (PE, Fig. 1a; median = 0), early secretory endometrium (ESE, Fig. 1b; median = 0), or late secretory endometrium (LSE, Fig. 1c; median = 0). We identified significant upregulation of VDR expression, with a median IRS value of 4.8 in the nuclei of glandular ovarian endometrial cells (*p* < 0.02, Fig. 1d) compared to normal endometrium in the three phases. We did not identify any significant differences in the expression of cytoplasmic VDR in either the normal endometrium or endometriosis (ovarian endometriosis median = 2; *p* = 0.053, PE median = 4, ESE median = 0, LSE median = 8.2). A summary of the staining results for VDR expression in the nuclei of the glandular cells is presented in Fig. 1e.

### AHR expression in the nuclei of glandular epithelial cells in tissue of normal endometrium and in endometriosis

**Fig. 2:**
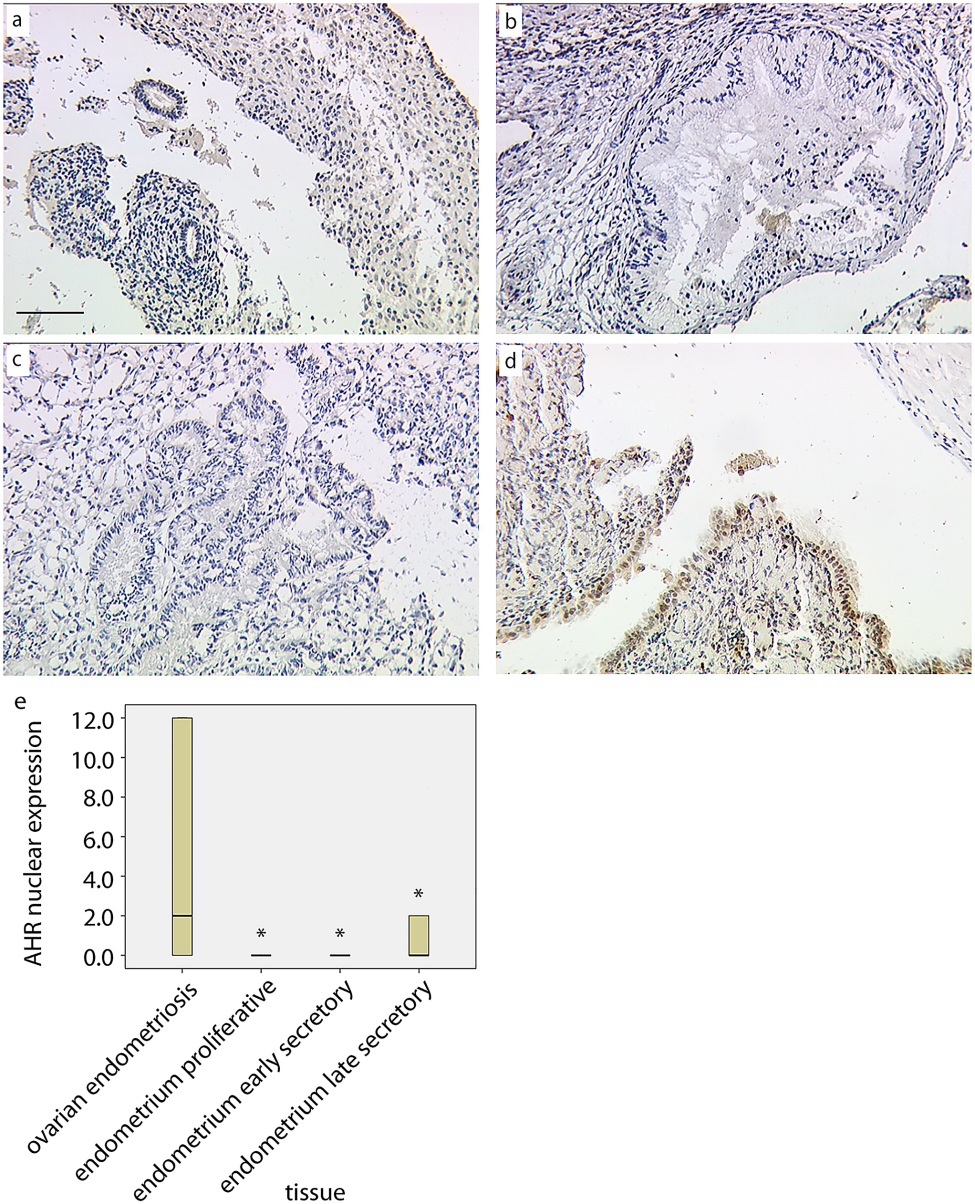
Expression of AHR in the nucleus of endometrial glands from proliferative (**a**), early secretory (**b**) and late secretory (**c**) endometrium as well as from ovarian endometriosis (**d**). Box and whiskers plot (**e**) of IRS scoring data for the different tissues (*significantly different versus “ovarian endometriosis”, Kruskal–Wallis test, *p* < 0.05). Bar = 100 µm

Similar to VDR expression, we were not able to observe expression of AHR in the nuclei of the glandular epithelial tissue (EP median = 0, ESE median = 0, LSE median = 0, Fig. 2a–c) in normal endometrium. We identified a significant enhanced AHR expression with a median IRS value of 1 in nuclei of glandular ovarian endometriosis (*p* < 0.03, Fig. 2d) compared to three phases of normal endometrial tissue. A summary of the staining results for AHR expression in the nuclei of the glandular cells is presented in Fig. 2e.

### AHR expression in the cytoplasm of glandular epithelial tissue of normal endometrium and in endometriosis

**Fig. 3:**
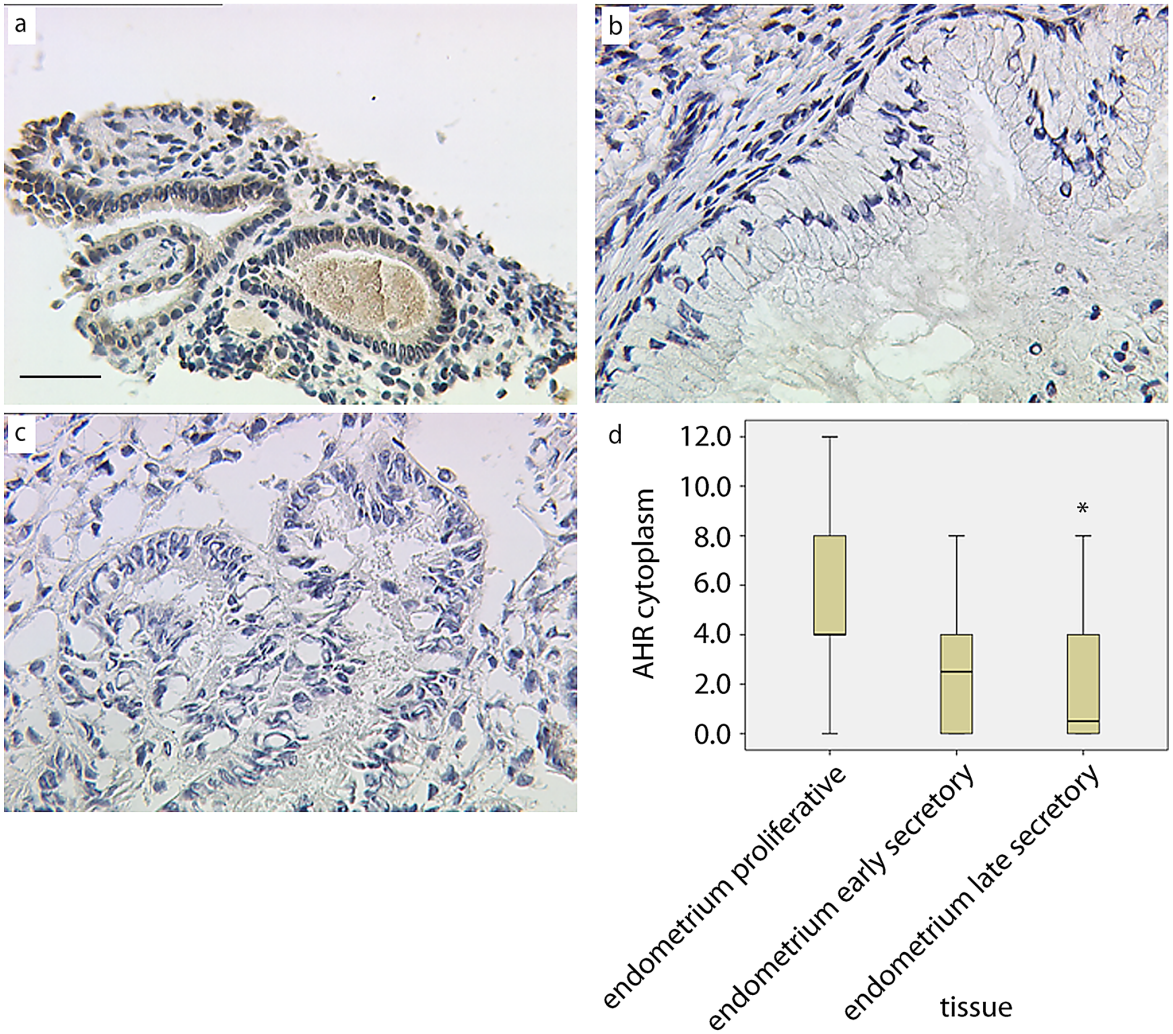
Expression of AHR in the cytoplasm of endometrial glands from proliferative (**a**), early secretory (**b**), and late secretory (**c**) endometrium. “Ovarian endometriosis” samples are provided as supplementary material (Supplementary Fig. 1) due to the lack of AHR expression in these tissues. Box and whiskers plot (**d**) of IRS scoring data for each tissue (*significantly different versus “proliferative endometrium”, Kruskal–Wallis test, *p* < 0.05). Bar = 50 µm

We identified a significant change in the expression of cytoplasmic AHR between the proliferative and late secretory endometrium. Indeed, cytoplasmic AHR seems to be upregulated in the proliferative endometrium (median = 4, Fig. 3a) compared to the late secretory endometrium (median = 0.4, Fig. 3b, *p* < 0.05). No significant differences were found for AHR expression in the cytoplasm of the early secretory endometrium (median = 2.3, Fig. 3c) compared to the proliferative and late secretory endometrium. No AHR expression was found in the cytoplasm of ovarian endometriosis samples (Supplementary Fig. 1). A summary of the staining results for AHR expression in the cytoplasm of the glandular cells is presented in Fig. 3d.

### Correlation between VDR and AHR expression in glandular cell nuclei of tissues in endometriosis

**Fig. 4:**
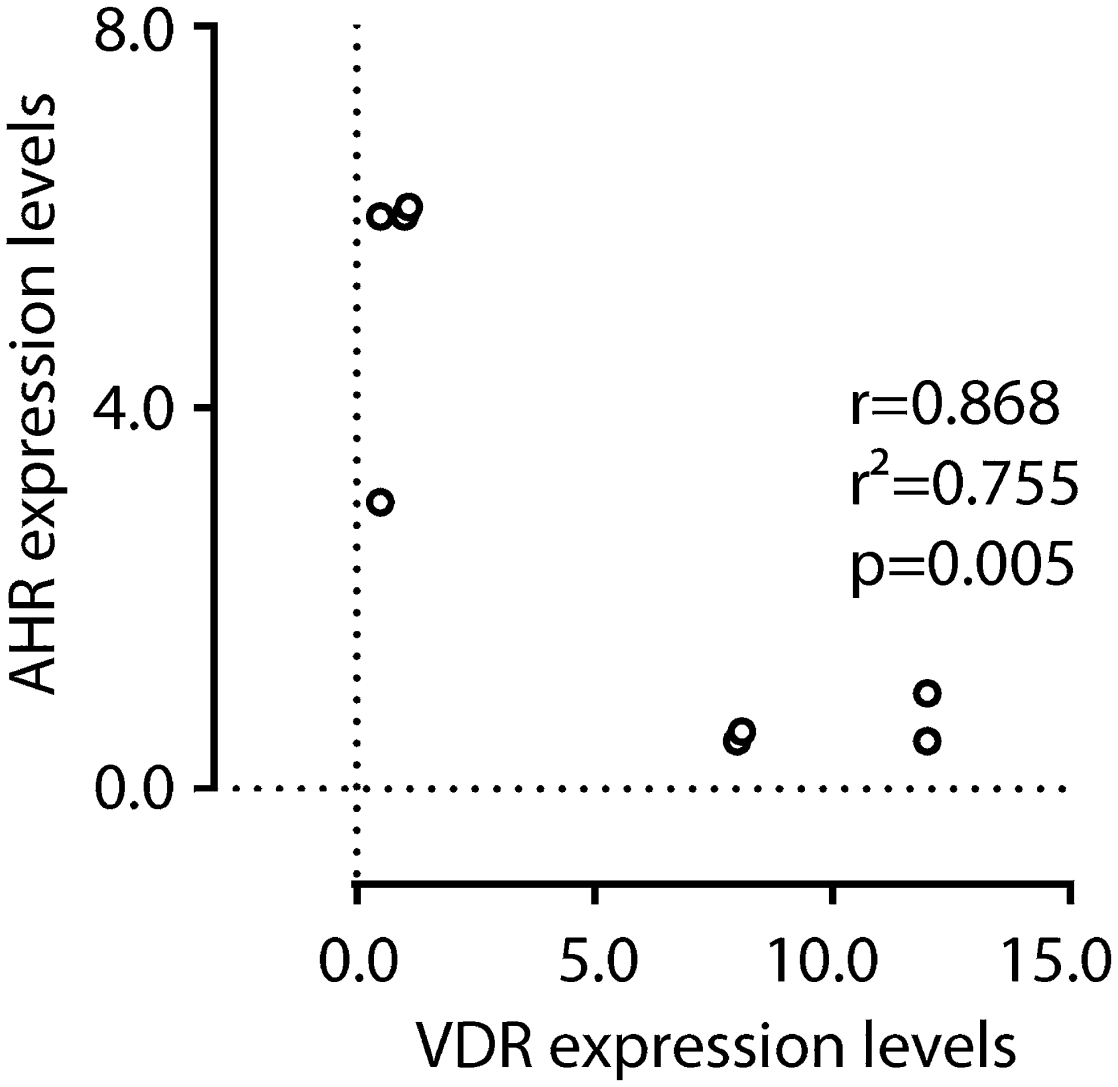
Negative correlation between VDR expression and AHR expression. Endometriosis tissue samples from different patients are plotted as a function of VDR and AHR IRS scores. Data correlation was evaluated by Pearson *r* test (*p* < 0.05)

We identified a negative correlation in the expression of VDR and AHR in the nuclei of the glandular cells (coefficient of correlation: −0.97, *p* = 0.007). As shown in Fig. 4, samples which showed high expression of VDR did not express AHR at an appreciable level. Conversely, samples with little VDR expression showed a high degree of AHR expression. Such differential expression may be interpreted as mutual exclusivity in the regulation of VDR and AHR: when one of these two receptors is highly expressed, the other one is similarly reduced.

### Stroma analysis shows no significant expression

We analyzed VDR and AHR expression in the stroma of the tissues, both for the three phases of the endometrium and for ovarian endometriosis. We considered only cells belonging to the stroma adjacent to glandular cells. We did not identify any significant expression for VDR and AHR in either the nuclei or cytoplasm of stromal cells (not shown).

## Discussion

VDR and AHR belong to the family of the nuclear receptors consisting of a heterogenous group of proteins that are targeted by a large set of fat-soluble molecules, hormones, vitamins and xenobiotics (Chawla et al. 2001). They mediate the expression of genes involved in a broad range of reproductive, developmental, metabolic and immune response functions (Becnel et al. 2015). Given that VDR and AHR were both previously implicated in endometriosis, in this study we investigated the nuclear and cytoplasmic expression of VDR and AHR in tissues from normal endometrium (in the three physiological phases: proliferative, early, and late secretory) and ovarian endometriosis. We found significantly higher expression of VDR and AHR in the nuclei of glandular cells derived from ovarian endometriosis compared to the three phases of the normal endometrium. Furthermore, we found that cytoplasmic AHR is upregulated specifically in the proliferative endometrium compared to late secretory endometrium but not early secretory endometrium. Spearman’s analysis revealed a negative correlation in the expression of VDR and AHR in the nuclei of the glandular cells from ovarian endometriosis, which led us to speculate that VDR and AHR expression is mutually exclusive in this condition.

### VDR is important for cell differentiation

Du et al. (2005) demonstrated the important role of VDR and its metabolites in regulating cell differentiation by acting on HOXA10 in human myelomonocytic cells and human endometrial stromal cells (Du et al. 2005). Human uterine endometrial cells and decidual cells synthesize 1,25-${(\mathrm{O}\mathrm{H})}_{2}$ ${\mathrm{D}}_{3}$ (Kachkache et al. 1993). Consequently, patients with pseudo-vitamin D deficiency (Glorieux et al. 1995), VDR knockout mice, and 1-α-hydroxylase knockout mice (Panda et al. 2001) show defective decidualization, inadequate uterine development, and anovulation, respectively. All these data confirm that vitamin D has an essential role in fertility, necessary for the differentiation of decidual cells. 1,25-${(\mathrm{O}\mathrm{H})}_{2}$ ${\mathrm{D}}_{3}$ also potently inhibits cellular proliferation and induces differentiation of myeloid leukemia cells (Du et al. 2005). Several genes are upregulated by 1,25-${(\mathrm{O}\mathrm{H})}_{2}$ ${\mathrm{D}}_{3}$ during myeloid differentiation including the HOXA family gene HOXA10. Du et al. (2005 demonstrated that 1,25-${(\mathrm{O}\mathrm{H})}_{2}$ ${\mathrm{D}}_{3}$ induces HOXA10 transcription through VDR binding to a vitamin D-response element (VDRE) in the HOXA10 gene 5′ region. HOXA genes have many important roles in the development of organisms, and HOXA10 expression is important for the development of the uterus (Taylor et al. 1997) and essential for endometrial development (Block et al. 2000), allowing uterine receptivity to implantation (Bagot et al. 2000). In summary, the authors state that vitamin D may induce differentiation of diverse tissues through activation of classic development modulators such as HOXA genes.

### AHR is important for cell proliferation

Many studies suggest that the effect of AHR ligands could be associated with their capacity to alter signal transduction pathways controlling cell proliferation and apoptosis (Ma and Whitlock 1996; Levine-Fridman et al. 2004). In particular, de Tomaso Portaz et al. (2015) investigated hexachlorobenzene-mediated cell proliferation and showed how this compound induced expression of the aryl hydrocarbon receptor in preneoplastic foci in the rat liver, illustrating its role as a mediator of ERK1/2 signaling (de Tomaso Portaz et al. 2015). ERK1/2 signaling is one of the most important pathways controlling the cell cycle and promoting cellular proliferation.

## Conclusions

The pathogenesis of endometriosis involves the formation of endometrial-like tissue outside the uterus, in areas where it should not be present. The endometrium is a very differentiated tissue, and the expression of VDR may contribute to the pathogenesis of endometriosis in terms of induction of endometrial-like differentiation in tissue where endometrial cells should not be present. The endometrium is also a proliferative tissue, and the expression of AHR may be important in the regulation of the cell cycle and induction of endometrial-like tissue. Thus, both mechanisms are implicated in the pathogenesis of endometriosis. Our data show an upregulation of AHR in proliferative endometrium compared to late secretory endometrium, which is consistent with a proliferation activity of AHR in the endometrium. Furthermore, our data suggest that expression of VDR and AHR is mutually exclusive in ovarian endometriosis. Such a phenomenon may be explained by a divergence between a more pro-differentiation fate mediated by VDR versus a more pro-proliferative fate induced by AHR, although more detailed studies are needed. Interestingly, a correlation between VDR expression and a woman’s age may also be revealed, as shown by plotting gene expression data against the age of patients (Supplementary Fig. 2). These data suggest that high VDR receptor expression, possibly due to vitamin D insufficiency, could occur as a function of age rather than the severity of endometriosis. However, these considerations must be confirmed by a clinical study with proper sample size, aiming to evaluate the age-related expression of specific molecules. Given the nature of our study, which retrospectively evaluated AHR and VDR expression in samples from the archives of the clinics, information available about patients, such as the proliferative phase of the endometrium, are limited. For instance, a biopsy of the uterine endometrium was not performed in the context of endometriosis surgery due to ethical issues, leading to missing information that must be taken as a limitation of the study.

Multiple pharmacological treatments for endometriosis have been suggested based on presumptive pathogenic mechanisms or hypothesized hormonal selectiveness (Vercellini et al. 2011). The current medical treatment has focused on the hormonal alteration of the menstrual cycle, with the major goal of producing a state of pseudo-pregnancy (Olive and Pritts 2001) through downregulation of the hypothalamic-pituitary-ovarian pathway (Valle and Sciarra 2003). New drugs and related targets have recently been proposed for the treatment of endometriosis. Some are more effective than others, but so far no definitive treatment is available (Guo 2008). Although there is no doubt that further, more in-depth studies are needed, our work has highlighted some characteristics of endometrial lesions by describing the differential expression of nuclear receptor VDR and AHR, providing more information on this heterogeneous disease. This study provides a possible starting point for developing more effective drugs specifically targeting VDR- or AHR-expressing cells in the context of endometriosis. Such drugs might act as antagonists downregulating pro-differentiation and proliferative signaling mediated by these receptors.

## Supplementary Information

Below is the link to the electronic supplementary material.

Supplementary file1 (TIF 2836 KB) Supplementary Figure 1: Negative staining of AHR expression in the cytoplasm of “ovarian endometriosis” samples. Picture is representative of four independent experiments; ×10 magnification; bar = 200 µm

Supplementary file2 (TIF 326 KB) Supplementary Figure 2: Linear regression analysis between VDR and AHR expression and patients’ age. VDR expression increases with patients’ age, but no such increase is seen for AHR expression (linear regression; *p* < 0.05)

## References

[CR1] Agic A, Xu H, Altgassen C, Noack F, Wolfler MM, Diedrich K, Friedrich M, Taylor RN, Hornung D (2007). Relative expression of 1,25-dihydroxyvitamin D3 receptor, vitamin D 1 alpha-hydroxylase, vitamin D 24-hydroxylase, and vitamin D 25-hydroxylase in endometriosis and gynecologic cancers. Reprod Sci.

[CR2] Bagot CN, Troy PJ, Taylor HS (2000). Alteration of maternal Hoxa10 expression by in vivo gene transfection affects implantation. Gene Ther.

[CR3] Becnel LB, Darlington YF, Ochsner SA, Easton-Marks JR, Watkins CM, McOwiti A, Kankanamge WH, Wise MW, DeHart M, Margolis RN, McKenna NJ (2015). Nuclear Receptor Signaling Atlas: Opening Access to the Biology of Nuclear Receptor Signaling Pathways. PLoS ONE.

[CR4] Berger U, Wilson P, McClelland RA, Colston K, Haussler MR, Pike JW, Coombes RC (1988). Immunocytochemical detection of 1,25-dihydroxyvitamin D receptors in normal human tissues. J Clin Endocrinol Metab.

[CR5] Block K, Kardana A, Igarashi P, Taylor HS (2000). In utero diethylstilbestrol (DES) exposure alters Hox gene expression in the developing mullerian system. FASEB J.

[CR6] Chawla A, Repa JJ, Evans RM, Mangelsdorf DJ (2001). Nuclear receptors and lipid physiology: opening the X-files. Science.

[CR7] Choi M, Makishima M (2009). Therapeutic applications for novel non-hypercalcemic vitamin D receptor ligands. Expert Opin Ther Pat.

[CR8] de Tomaso Portaz AC, Caimi GR, Sanchez M, Chiappini F, Randi AS, Kleiman de Pisarev DL, Alvarez L (2015). Hexachlorobenzene induces cell proliferation, and aryl hydrocarbon receptor expression (AhR) in rat liver preneoplastic foci, and in the human hepatoma cell line HepG2. AhR is a mediator of ERK1/2 signaling, and cell cycle regulation in HCB-treated HepG2 cells. Toxicology.

[CR9] Donnez J, Squifflet J, Pirard C, Jadoul P, Wyns C, Smets M (2002). The efficacy of medical and surgical treatment of endometriosis-associated infertility and pelvic pain. Gynecol Obstet Invest.

[CR10] Du H, Daftary GS, Lalwani SI, Taylor HS (2005). Direct regulation of HOXA10 by 1,25-(OH)2D3 in human myelomonocytic cells and human endometrial stromal cells. Mol Endocrinol.

[CR11] Glorieux FH, Arabian A, Delvin EE (1995). Pseudo-vitamin D deficiency: absence of 25-hydroxyvitamin D 1 alpha-hydroxylase activity in human placenta decidual cells. J Clin Endocrinol Metab.

[CR12] Guo SW (2008). Emerging drugs for endometriosis. Expert Opin Emerg Drugs.

[CR13] Guyton KZ, Kensler TW, Posner GH (2003). Vitamin D and vitamin D analogs as cancer chemopreventive agents. Nutr Rev.

[CR14] Holick MF (2003). Vitamin D: A millenium perspective. J Cell Biochem.

[CR15] Kachkache M, Rebut-Bonneton C, Demignon J, Cynober E, Garabedian M (1993). Uterine cells other than stromal decidual cells are required for 1,25-dihydroxyvitamin D3 production during early human pregnancy. FEBS Lett.

[CR16] Koeffler HP, Aslanian N, O'Kelly J (2005). Vitamin D(2) analog (Paricalcitol; Zemplar) for treatment of myelodysplastic syndrome. Leuk Res.

[CR17] Lemire JM, Archer DC, Beck L, Spiegelberg HL (1995). Immunosuppressive actions of 1,25-dihydroxyvitamin D3: preferential inhibition of Th1 functions. J Nutr.

[CR18] Levine-Fridman A, Chen L, Elferink CJ (2004). Cytochrome P4501A1 promotes G1 phase cell cycle progression by controlling aryl hydrocarbon receptor activity. Mol Pharmacol.

[CR19] Ma Q, Whitlock JP (1996). The aromatic hydrocarbon receptor modulates the Hepa 1c1c7 cell cycle and differentiated state independently of dioxin. Mol Cell Biol.

[CR20] Missmer SA, Cramer DW (2003). The epidemiology of endometriosis. Obstet Gynecol Clin North Am.

[CR21] Nagpal S, Na S, Rathnachalam R (2005). Noncalcemic actions of vitamin D receptor ligands. Endocr Rev.

[CR22] Nebert DW, Roe AL, Dieter MZ, Solis WA, Yang Y, Dalton TP (2000). Role of the aromatic hydrocarbon receptor and [Ah] gene battery in the oxidative stress response, cell cycle control, and apoptosis. Biochem Pharmacol.

[CR23] Ohtake F, Takeyama K, Matsumoto T, Kitagawa H, Yamamoto Y, Nohara K, Tohyama C, Krust A, Mimura J, Chambon P, Yanagisawa J, Fujii-Kuriyama Y, Kato S (2003). Modulation of oestrogen receptor signalling by association with the activated dioxin receptor. Nature.

[CR24] Olive DL, Pritts EA (2001). Treatment of endometriosis. N Engl J Med.

[CR25] Panda DK, Miao D, Tremblay ML, Sirois J, Farookhi R, Hendy GN, Goltzman D (2001). Targeted ablation of the 25-hydroxyvitamin D 1alpha -hydroxylase enzyme: evidence for skeletal, reproductive, and immune dysfunction. Proc Natl Acad Sci USA.

[CR26] Ravid A, Koren R (2003). The role of reactive oxygen species in the anticancer activity of vitamin D. Recent Res Cancer Res.

[CR27] Remmele W, Stegner HE (1987). Recommendation for uniform definition of an immunoreactive score (IRS) for immunohistochemical estrogen receptor detection (ER-ICA) in breast cancer tissue. Pathologe.

[CR28] Rier SE, Martin DC, Bowman RE, Dmowski WP, Becker JL (1993). Endometriosis in rhesus monkeys (Macaca mulatta) following chronic exposure to 2,3,7,8-tetrachlorodibenzo-p-dioxin. Fundam Appl Toxicol.

[CR29] Safe S, Lee SO, Jin UH (2013). Role of the aryl hydrocarbon receptor in carcinogenesis and potential as a drug target. Toxicol Sci.

[CR30] Sayegh L, Fuleihan Gel H, Nassar AH (2014). Vitamin D in endometriosis: a causative or confounding factor?. Metabolism.

[CR31] Taylor HS, Vanden Heuvel GB, Igarashi P (1997). A conserved Hox axis in the mouse and human female reproductive system: late establishment and persistent adult expression of the Hoxa cluster genes. Biol Reprod.

[CR32] Valle RF, Sciarra JJ (2003). Endometriosis: treatment strategies. Ann N Y Acad Sci.

[CR33] Varma R, Rollason T, Gupta JK, Maher ER (2004). Endometriosis and the neoplastic process. Reproduction.

[CR34] Vercellini P, Crosignani P, Somigliana E, Vigano P, Frattaruolo MP, Fedele L (2011). 'Waiting for Godot': a commonsense approach to the medical treatment of endometriosis. Hum Reprod.

[CR35] Vigano P, Lattuada D, Mangioni S, Ermellino L, Vignali M, Caporizzo E, Panina-Bordignon P, Besozzi M, Di Blasio AM (2006). Cycling and early pregnant endometrium as a site of regulated expression of the vitamin D system. J Mol Endocrinol.

[CR36] Zondervan KT, Becker CM, Missmer SA (2020). Endometriosis. N Engl J Med.

