# Supplementary figures and images for: Nuclear expression of VDR and AHR is mutually exclusive in glandular cells in endometriosis
Source: Histochem Cell Biol. 2021 Jun 21;156(4):391–9. doi: 10.1007/s00418-021-02005-9 (PMC8550147; doi:10.1007/s00418-021-02005-9)

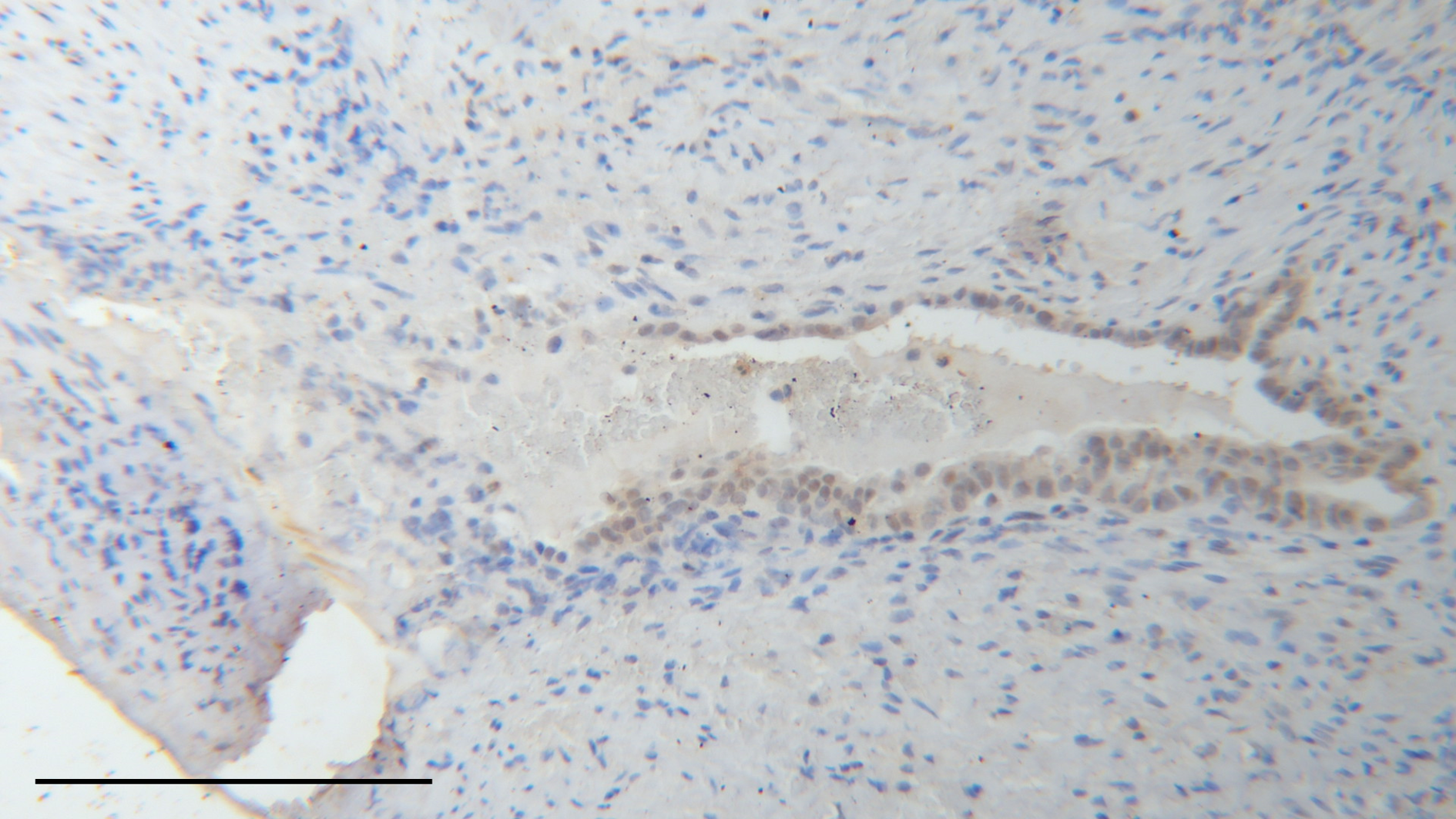

Supplement: Supplementary file 1 — Supplementary file1 (TIF 2836 KB) Supplementary Figure 1: Negative staining of AHR expression in the cytoplasm of “ovarian endometriosis” samples. Picture is representative of four independent experiments; ×10 magnification; bar = 200 µm [file 418_2021_2005_MOESM1_ESM.tif]

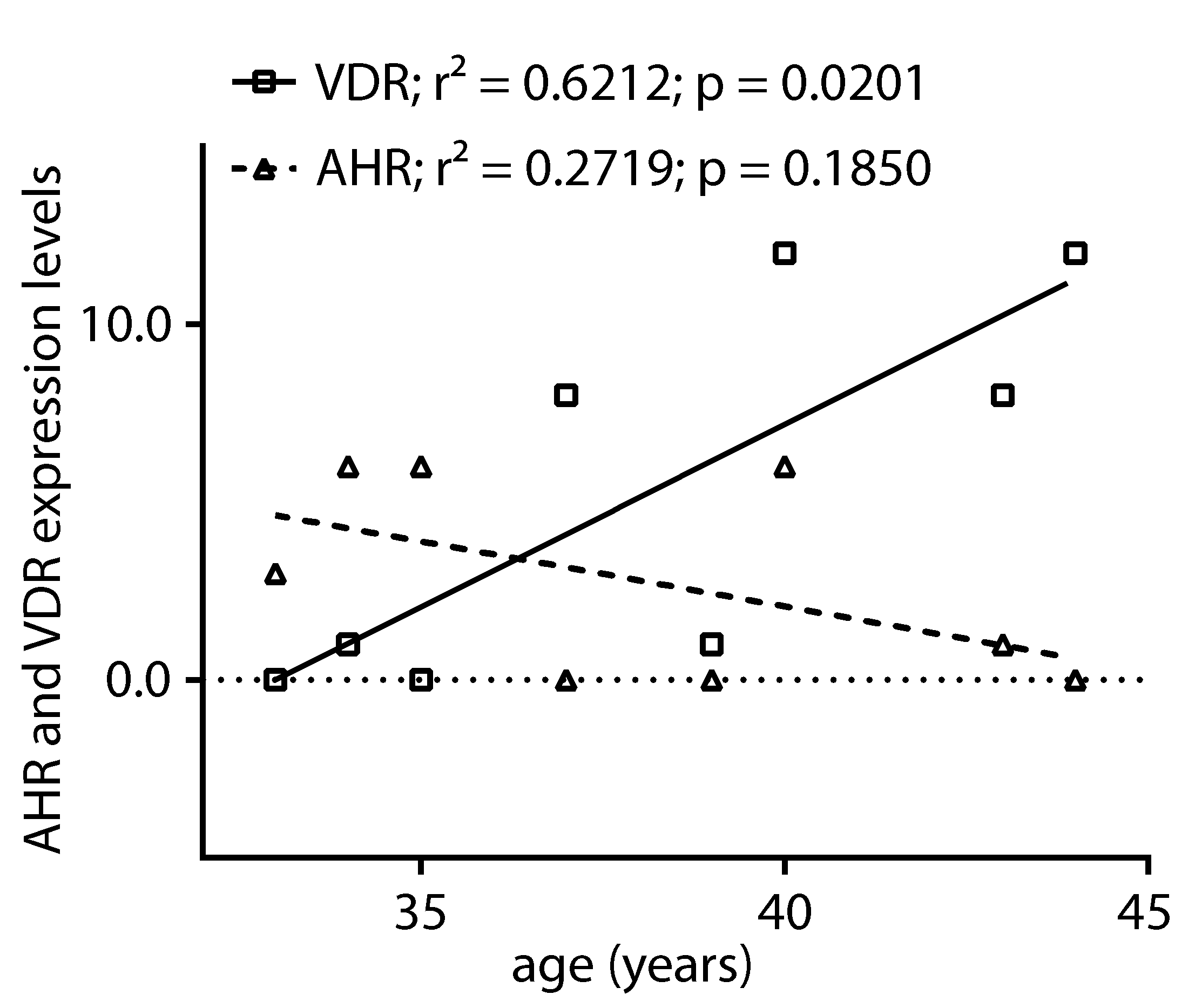

Supplement: Supplementary file 2 — Supplementary file2 (TIF 326 KB) Supplementary Figure 2: Linear regression analysis between VDR and AHR expression and patients’ age. VDR expression increases with patients’ age, but no such increase is seen for AHR expression (linear regression; p < 0.05) [file 418_2021_2005_MOESM2_ESM.tif]
